# Molecular detection of tick-borne pathogens in ticks collected from pets in selected mountainous areas of Tatra County (Tatra Mountains, Poland)

**DOI:** 10.1038/s41598-020-72981-w

**Published:** 2020-09-28

**Authors:** Anna Kocoń, Marek Asman, Magdalena Nowak-Chmura, Joanna Witecka, Małgorzata Kłyś, Krzysztof Solarz

**Affiliations:** 1 Institute of Biology, Department of Zoology, Pedagogical University of Kraków, Podchorążych 2, 30-084 Kraków, Poland; 2 Department of Parasitology, Faculty of Pharmaceutical Sciences in Sosnowiec, Medical University of Silesia, Jedności 8, 41-218 Sosnowiec, Poland; 3 Institute of Biology, Department of Ecology and Environmental Protection, Pedagogical University of Kraków, Podchorążych 2, 30-084 Kraków, Poland

**Keywords:** Zoology, Entomology, Ecological epidemiology, Parasitology, Pathogens

## Abstract

The mountainous and foothill areas, in which the city of Zakopane, the capital of Tatra County, is located are characterized by continuous weather changes, lower air temperature, persistent snow cover, and poorer vegetation than in the lowlands. *Ixodes ricinus* and *Ixodes hexagonus* are vectors of tick-borne diseases and play an important role in the persistence of tick-borne diseases. The aim of the study was to determine the risk of exposure of domestic cats and dogs to the attacks of Ixodid ticks, to tick-borne infections with *Borrelia burgdorferi* sensu lato, *Anaplasma phagocytophilum, Babesia microti* and *Toxoplasma gondii* in the city of Zakopane and the surrounding area. In 2017–2018 ticks were collected from a total of 10 domestic cats and 88 domestic dogs. Selected pathogens of tick-borne diseases were detected by PCR and nested PCR. The study material contained 119 *I. ricinus* and 36 *I. hexagonus*. The molecular examinations showed the presence of *A. phagocytophilum* in 3.8%, *B. microti* in 24.5% and *T. gondii* in 4.5% of the all ticks. In addition, in the study area, there is a high potential risk of tick-borne infection by *B. microti*, and a low potential risk of exposure to *A. phagocytophilum* and *T. gondii* infection.

## Introduction

The geographical location of Zakopane, numerous tourist and walking routes, a large number of wooden architecture monuments, landscape diversity, and climatic and medicinal values are undoubtedly the advantages of the city, which is visited by more and more tourists from Poland, Europe and all over the world every year. The fauna of ticks (Acari: Ixodidae) in Tatra County is hardly known. Due to the adverse habitat and climatic conditions, such as high weather variability, low annual air temperatures and ever lower air temperature as altitude increases, as well as frequent rainfall, persistent snow cover, and consequently poor vegetation, these areas are not a convenient habitat for ticks. Moreover, the Tatra National Park is a protected area that makes it difficult for researchers to access.

It is known that *Ixodes ricinus* (Linnaeus, 1758) is the most common among the 19 tick species found in the Polish fauna. The species attacks numerous mammals, birds and reptiles. *I. ricinus* can be a vector and/or reservoir of many pathogens, including *Borrelia burgdorferi* s. l., *Anaplasma phagocytophilum*, *Babesia* sp.^1–3^. The second tick species widespread throughout Europe, and therefore probably also in Poland is *Ixodes hexagonus* (Leach, 1815), which feeds mainly on hedgehogs, weasels, foxes and domestic dogs^4–7^. Like *I. ricinus*, the tick can be a vector and/or reservoir of many pathogens, e.g. *B*. *burgdorferi* s. l., *A*. *phagocytophilum* and *Babesia* sp.^7^. Domestic cats and dogs can be hosts of both tick species^8^. In Poland, these domestic animals are mainly attacked by five species, i.e. *I*. *ricinus*, *Ixodes crenulatus* (Koch, 1844), *I*. *hexagonus*, *Ixodes rugicollis* (Schulze and Schlottke, 1929) and *Dermacentor reticulatus* (Fabricius, 1794)^9^.

Due to the great medical and veterinary importance of ticks and close contact between human and domestic animals, an attempt has been made to determine the exposure of cats and dogs to tick infection, as well as to a potential tick-borne infection with *B*. *burgdorferi* s. l. and *A*. *phagocytophilum,* as well as to the infection of *Babesia microti* and *Toxoplasma gondii* in the selected areas of Tatra County. The approach of gathering data on the distribution of (zoonotic) vector-borne diseases through a veterinary survey is consistent with the ‘One Health’ concept^10^.

## Materials and methods

Zakopane, is the highest located city in Poland (49° 18′ N; 19° 57′ E), situated in the Podtatrzanski Trench, between the Tatra Mountains and the Beskids, in one of the largest mountain ranges in Europe—the Carpathian Mountains. The urban part stretches from 750 to 1000 m a.s.l., the administrative boundaries of the city contain a part of the Tatra Mountains with the highest point—the peak of Swinica (2301 m a.s.l.), as well as Gubalowka (1120 m a.s.l.), and the central point of Zakopane is located at an altitude of 838 m a.s.l.

The research material was obtained from domestic cats and dogs in 2017–2018 from March to September in cooperation with the veterinary clinic in Zakopane. The ticks were collected from animals using tweezers and placed in tubes with 70% ethyl alcohol. In addition, after the collection, an original form was completed with the following information: date of collection, animal breed, sex, age and the city of collection. Then, individual ticks were determined for genus, species and developmental stage. The keys by Siuda^1^ and Nowak-Chmura^7^ were used to identify the ticks. Next, molecular testing for the presence of selected pathogens was performed. DNA was isolated from 155 ticks using the ammonium hydroxide method^11^. Then, the concentration was measured spectrophotometrically at the wave length of 260/280 nm. The pathogens in the material were detected by PCR and nested PCR. For detection of *B*. *burgdorferi* s.l., Maximo DFS-Plus polymerase (GeneOn, Germany) and flagellin gene specific primers were used^12^. Taq DNA polymerase (EURx, Poland) and two pairs of specific primers for the 16S rRNA gene were applied to detect *A. phagocytophilum*^13^. The protozoa of *B. microti* and *T. gondii* were detected with two pairs of specific primers for the 18S rRNA gene and Maximo DFS-Plus polymerase (GeneOn, Germany) and for the B1 gene with the use of Taq DNA polymerase (EURx, Poland)^14,15^. The amplification and re-amplification products were separated electrophoretically on 2% ethidium bromide stained agarose gels. Then, the gels were visualized in ultraviolet light. The following reaction products were treated as positive: 482 base pairs [bp] for *B*. *burgdorferi* s. l., 932 bp and 546 bp for *A. phagocytophilum*, 238 bp and 154 bp for *B. microti* and 531 bp for *T. gondii*. Statistical analysis was performed using CSS-Statistica for Windows 10. Statistical significance was accepted at a p value of less than 0.05. The results were analysed using an χ^2^ tests.

### Ethical approval

We declare that all testing methods have been carried out in accordance with the relevant guidelines and regulations. We declare that all experimental protocols have been approved by the Medical University of Silesia in Katowice and Pedagogical University in Cracow.

### Informed consent

Each dog and cat owner has agreed to collect material (ticks) from their animals and give informed consent to publish the results of the collected material.

## Results

**Figure 1:**
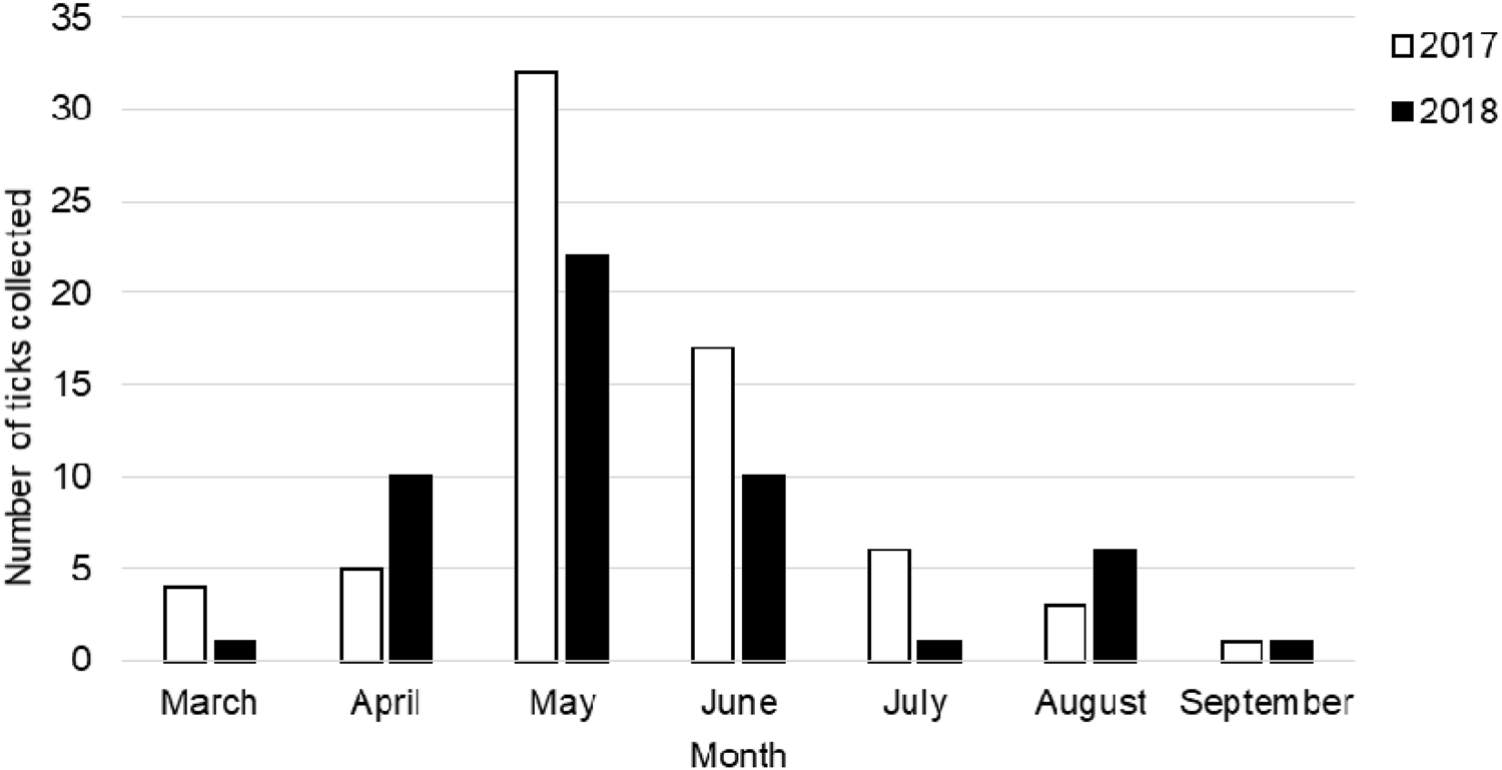
The total number of *Ixodes ricinus* ticks collected from domestic animals in Zakopane in 2017–2018.

A total of 155 ticks were collected from 10 domestic cats and 88 domestic dogs. *I. ricinus* was the dominant tick species, with 119 females collected from 90 animals, including 8 cats and 82 dogs. 36 *I. hexagonus* ticks were also collected, including 15 larvae, 13 nymphs and 8 females. The presence of this tick species has been demonstrated in 8 animals, including 2 cats and 6 dogs. The infestation by *I. ricinus* usually occurred in May, while it was least often reported in March and September (Fig. 1).

**Figure 2:**
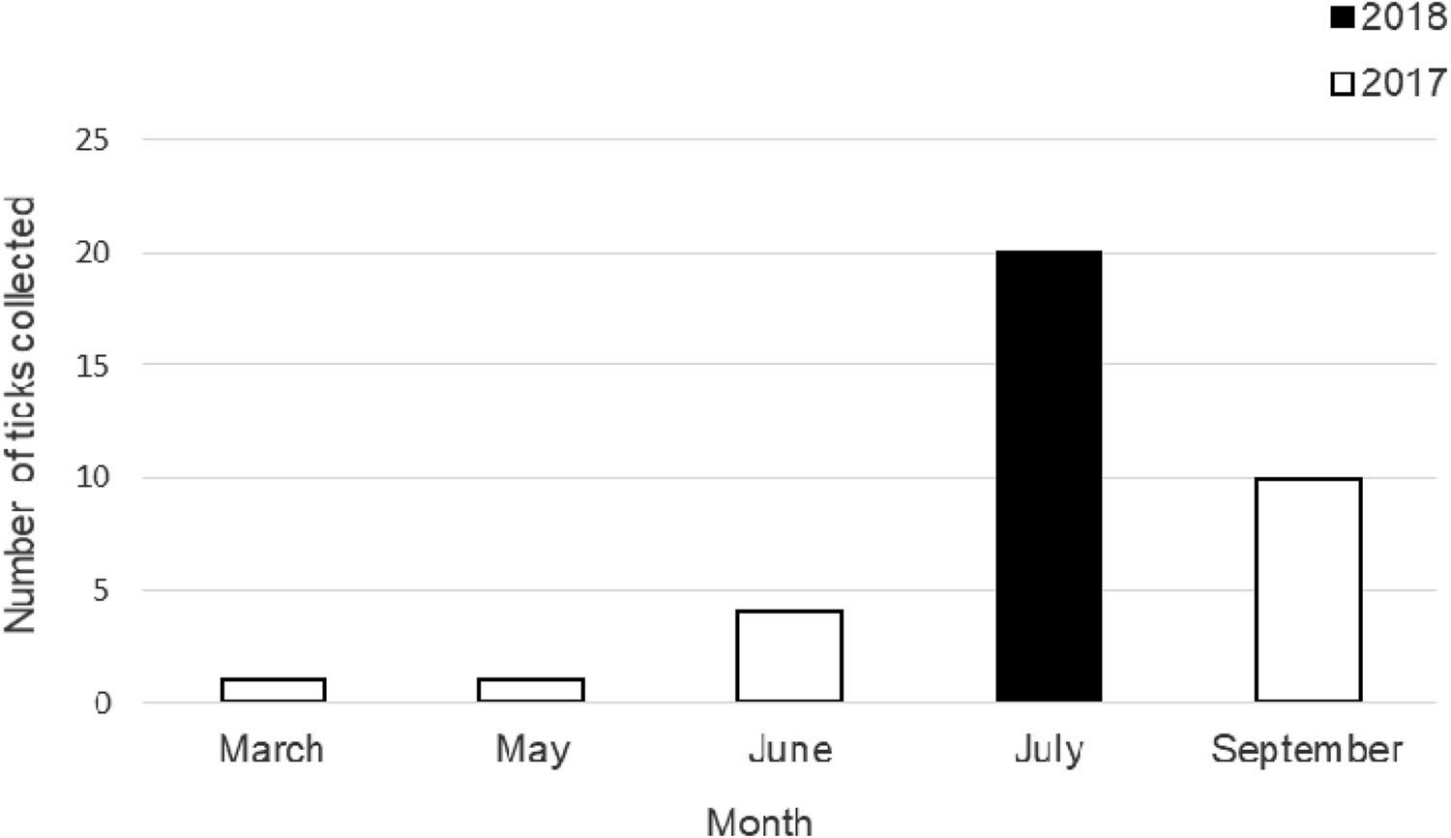
The total number of *Ixodes hexagonus* ticks collected from domestic animals in Zakopane in 2017–2018.

*I. hexagonus* species usually invaded in July, and it was least frequently found in March and May (Fig. 2).

In total, the pathogens were found in 26.0% of *I. ricinus* individuals and in 50.0% of *I. hexagonus* ticks. This difference was statistically significant (Yates corrected χ^2^ = 11.23; p = 0.0008). *A. phagocytophilum* was found only in 3.4% of female *I. ricinus* ticks. On the other hand, protozoa of *B. microti* and *T. gondii* were reported in both tick species. *B. microti* was reported in a much higher percentage of *I. ricinus* and *I. hexagonus* individuals than *T. gondii* (Table 1). *B*. *burgdorferi* s. l. were not shown in the all study material. In addition, the coexistence of *A. phagocytophilum* and *B. microti*, as well as *A. phagocytophilum* and *T. gondii* was demonstrated in *I. ricinus* females (Table 1). In general, all ticks collected from cats and dogs were statistically significantly more often infected with *B. microti* than with *T. gondii* and *A. phagocytophilum* (Yates corrected χ^2^; p ≤ 0.00001, in all cases).

**Table 1:** The total number and percentage of *Ixodes ricinus* and *Ixodes hexagonus* ticks infected with *Anaplasma phagocytophilum, Babesia microti* and *Toxoplasma gondii* collected from domestic cats and dogs in Zakopane and the surrounding area.

Tick species	Total number of studied ticks	1 pathogen			2 pathogens	
		*Anaplasma phagocytophilum*	*Babesia microti*	*Toxoplasma gondii*	*Anaplasma phagocytophilum* + *Babesia microti*	*Anaplasma phagocytophilum* + *Toxoplasma gondii*
*Ixodes ricinus*	119	4 (3.4%)	29 (24.3%)	4 (3.7%)	1 (0.8%)	1 (0.8%)
*Ixodes hexagonus*	36	0 (0.0%)	8 (22.32%)	2 (5.5%)	0 (0.0%)	0 (0.0%)
Total	155	4 (2.6%)	37 (23.8%)	6 (3.9%)	1 (0.6%)	1 (0.6%)

Mainly larvae of *I. hexagonus* contained *B. microti* (46.7%), whereas *T. gondii* was found in a similar percentage in both juvenile forms (Table 2). The difference was statistically significant (Yates corrected χ^2^ = 38.58 and 36.21 for larvae and nymphs, respectively; p ≤ 0.00001, in both cases).

**Table 2:** The total number and percentage of developmental stages of *Ixodes hexagonus* ticks infected with *Babesia microti* and *Toxoplasma gondii* collected from domestic cats and dogs in Zakopane and the surrounding area.

Developmental stage	Total number of studied ticks	1 pathogen	
		*Babesia microti*	*Toxoplasma gondii*
Larva	15	7 (46.7%)	1 (6.7%)
Nymph	13	0 (0.0%)	1 (7.7%)
Female	8	1 (12.5%)	0 (0.0%)
Total	36	8 (22.2)	2 (5.5%)

Generally, *I*. *hexagonus* collected from cats and dogs were statistically significantly more often infected with *B. microti* than with *T. gondii* (Yates corrected χ^2^ = 10.96; p = 0.0009).

*Babesia microti* was found in 28.1% of the ticks collected from dogs. *A. phagocytophilum* and *T. gondii* were found in 3.9% of the *I. ricinus* females (Table 3). This difference was statistically significant (Yates corrected χ^2^ = 19.68; p ≤ 0.00001). In addition, single cases of *A. phagocytophilum* and *B. microti* as well as *A. phagocytophilum* and *T. gondii* were reported in the ticks collected from dogs (Table 3).

**Table 3:** The number and percentage of *Ixodes ricinus* females infected with *Anaplasma phagocytophilum, Babesia microti* and *Toxoplasma gondii* collected from dogs in Zakopane and the surrounding area.

	Total number of studied ticks	1 pathogen			2 pathogens	
		*Anaplasma phagocytophilum*	*Babesia microti*	*Toxoplasma gondii*	*Anaplasma phagocyto*–*philum* + *Babesia microti*	*Anaplasma phagocytophilum* + *Toxoplasma gondii*
Females	103	4 (3.9%)	29 (28.1%)	4 (3.9%)	1 (1.0%)	1 (1.0%)
Total	103	4 (3.9%)	29 (28.1%)	4 (3.9%)	1 (1.0%)	1 (1.0%)

As for *I. hexagonus* ticks collected from cats, *B. microti* was shown in 54% of the nymphs and in 33.3% of the females. It should be emphasized that this difference was statistically significant (Yates corrected χ^2^ = 8.14; p = 0.0043). *T. gondii* was demonstrated in 7.1% of the larvae and in 7.7% of the nymphs of this tick species. Generally, hedgehog ticks collected from cats were statistically significantly more often infected with *B. microti* than with *T. gondii* (Yates corrected χ^2^ = 12.79; p = 0.0003).

## Discussion

Research on the presence of ticks in domestic animals, especially cats and dogs, is carried out around the world, including Europe. In this study *I*. *ricinus* was the predominant tick species infesting domestic dogs and cats, followed by the *I*. *hexagonus*. This is analogy with other studies in Europe^5,16–19^. The research conducted so far in the areas of southern, south-eastern and central Poland on the occurrence of ticks in domestic cats and dogs confirmed that dogs are usually infested by *I. ricinus*. Moreover, it has been shown that, in addition to the species mentioned above, other tick species may occasionally infest these animals. These include *I*. *hexagonus*, *D*. *reticulatus*, *I*. *crenulatus*^20–22^. In Poland, however, studies on the frequency of tick infestation of domestic cats have shown *I. ricinus, I. hexagonus, I. rugicollis* and *Ixodes apronophorus* (Schulze, 1924) to be main attackers with *I. ricinus* definitely dominating in numbers^8,23,24^. To date, three tick species have been found in Zakopane and the Tatra National Park: *I. ricinus, I. hexagonus* and *I. trianguliceps*^25^. Officially, the first *I. hexagonus* was collected in the Tatra National Park from the red vole (*Myodes glareolus*) by Jan Rafalski in 1964^1^. *I*. *trianguliceps* is a species associated with rodents. In 1980, however, Haitlinger noted a single *I*. *trianguliceps* larvae feeding on the common weasel (*Mustela nivalis*)^26^. Later, an individual of this species was collected from the red vole (*Myodes glareolus*)^25^. The studies confirmed the occurrence of *I. ricinus* and *I. hexagonus* in Zakopane and the surrounding area and the possibility of infection of domestic cats and dogs. Furthermore, it has been confirmed that *I. ricinus* is the species most often attacking domestic animals. Selected regions of the Polish Carpathians, including the Island Beskids, are areas of the common occurrence of the tick *I*. *ricinus*, recognized in medical and veterinary sense as the most dangerous tick in the Polish fauna of these parasites, and are also the habitat of other tick species, including *Carios vespertilionis*, *Ixodes lividus*, *Ixodes simplex*, *Ixodes trianguliceps*, *Ixodes rugicollis*, *Ixodes hexagonus*, *Ixodes vespertilionis*, *Ixodes crenulatus*^6,27–29^. In the Czech Republic, research was undertaken to check for the occurrence of tick-borne disease pathogens among *I*. *ricinus* in mountain areas and it was found that this tick species was recorded even up to a height of 1270 m a. s. l.^30^. Slovak tick researchers suggest that under the influence of global warming the upper limit of occurrence of these parasites is changing and now *I*. *ricinus* ticks can be found even at an altitude of up to 1460 m a.s.l. and *I*. *hexagonus* up to 1800 m a. s. l^31^. In 2004 and 2006–2011 studies were carried out to check the expansion of ticks to higher altitudes in the ecosystem of Little Fatra (northern Slovakia) and their infection *B*. *burgdorferi* s.l. The number of infected ticks decreased from 38.5% at the lowest altitude to 4.4% at the highest altitude^32^.

Molecular tests for *B. burgdorferi* s. l., *A. phagocytophilum, B. microti* and *T. gondii* showed that in both tick species most individuals were infected with *B. microti*. *T. gondii* was found in a significantly lower percentage of *I. ricinus* and *I. hexagonus* ticks. The values obtained are much lower than those received by Asman et al.^33^ in Tarnowskie Góry County. The researchers showed the presence of *B. microti* in 42.6% of the individuals, while *T. gondii* was in 98% of the ticks. In addition, these protozoa have been demonstrated in ticks collected from both cats and dogs^33^. Contrary to the studies conducted by Asman et al.^33^, *B. microti* and *T. gondii* have been shown in *I. ricinus* ticks collected only from dogs, and in *I. hexagonus* species collected only from cats. However, like in the analyses of ticks conducted in Tarnowskie Góry County, both these protozoan species were found mainly in *I. ricinus* females. This confirms the thesis that, apart from the nymph, this developmental stage is the main epidemiological threat for protozoa^33^. Ticks may play a large role in the transmission of *T*. *gondii*, but this requires further research.

*Anaplasma phagocytophilum* was found in a much smaller percentage of *I. ricinus* individuals and was not reported in *I. hexagonus*. The values are much lower than those obtained by Król et al.^8^ who demonstrated the presence of this rickettsia in 21.3% of *I. ricinus* ticks and in 8.1% of *I. hexagonus* individuals collected from cats and dogs in the agglomeration of Wroclaw. Other research conducted in south-eastern Poland also showed a high percentage of *I. ricinus* ticks infected with *A. phagocytophilum*^24^. On the other hand, the values presented in this work are only slightly higher than those obtained by Zygner et al.^21^ in central Poland. Similar studies conducted in several European countries on the occurrence of tick-borne pathogens, including *A. phagocytophilum*, in ticks collected from domestic cats and dogs, also showed a higher percentage of *I. ricinus* individuals infected with *A. phagocytophilum*^19^. The studies carried out in the Netherlands revealed a twice lower percentage of *I. ricinus* ticks infected with this rickettsia than in presented work, while the presence of *A. phagocytophilum* in *I. hexagonus* individuals was significantly higher^5^. There are also cases of *Borrelia burgdorferi* s. l. in ticks collected from domestic animals. The research conducted by Schreiber et al.^17^ in Germany showed the presence of this spirochete in 11.6% of *I. ricinus* and in 11.2% of *I. hexagonus* ticks collected from cats and dogs^17^. Studies conducted in The Netherlands and Belgium showed the presence of *B. burgdorferi* s. l. in 7.2% and 10.1% of *I. ricinus* individuals, respectively^5,16^. Several years of research conducted in urban areas of the Carpathian regions of Slovakia and Poland and their peripheral part, showed that specific *Borrelia burgdorferi* s. l. IgG antibodies, were found in 50% of 256 dogs, 6.9% of 29 cats from East Slovakia (Inner West Carpathian) and 42.6% of 68 dogs from the Lublin district^34^. Similar studies carried out in several European countries have shown the presence of this bacterium mainly in *I. ricinus* ticks collected from cats. Studies conducted in France also demonstrated the presence of *B. afzelii* in *I. hexagonus* tick collected from a cat^19^. The research carried out in Poland has shown that the occurrence of this bacterium in ticks collected from animals may range from 6.2% in *I. ricinus* in central Poland to 22.8% in *D. reticulatus* in south-eastern Poland^21,24^. 'The absence of *B*. *burgdorferi* s.l. in ticks collected from domestic animals in Tatra County may result from the fact that an increase of altitude is related to a decrease in the number of ticks infected with this bacterium, as shown by Taragelova et al.^32^.

It is commonly known that ticks can be vectors and/or reservoirs of more than one pathogen. There are cases of co-occurrence of two or three pathogens in *I. ricinus* ticks collected from vegetation, but the percentage of such ticks in the population is very low^12,35–37^. Also, there are cases of such coinfection in ticks collected from domestic animals^8,33^. The research conducted in Tarnowskie Góry County by Asman et al.^33^ showed the co-occurrence of *B. microti* and *T. gondii* in more than 40% of *I. ricinus* ticks collected from cats and dogs. Moreover, co-infection was reported mainly in female *I. ricinus* ticks collected from dogs. On the other hand, Król et al.^8^ demonstrated the coexistence of 2 or even 3 pathogens in a single *I. ricinus* tick, with *A. phagocytophilum* and *Rickettsia spp.* most frequently found in co-infection. The ticks collected from dogs in Tatra County showed coexistence of *A. phagocytophilum* and *B. microti* as well as *A. phagocytophilum* and *T. gondii* in only two *I. ricinus* females. However, coexistence of these pathogens was not observed in the ticks collected from cats, which may result from a small number of ticks collected from these animals.

## Conclusions

The research indicates that potentially unfavourable environmental conditions for ticks in Tatra County do not prevent domestic cats and dogs from a high risk of exposure to the infestation by *I. ricinus* and *I. hexagonus* ticks. In addition, the study revealed a potentially high risk of tick-borne infection of *B. microti* and a low risk of exposure to a tick-borne *A. phagocytophilum* infection and *T. gondii* invasion in the study area. Moreover, the results show possible coexistence of *A. phagocytophilum* and both *B. microti* and *T. gondii* in *I. ricinus* species. However, it cannot be excluded the possibility that some individuals may have been pathogen-positive because of feeding on an infected (asymptomatic) animal. The lack of *Borrelia burgdorferi* s. l. in the material may be due to a generally low percentage of ticks infected with this bacterium in the study area, and this may result from the geographical location.
